# A Brief History of Cell Culture: From Harrison to Organs-on-a-Chip

**DOI:** 10.3390/cells13242068

**Published:** 2024-12-15

**Authors:** Lincoln Gozzi Moro, Lucas Pires Guarnier, Maurício Fogaça Azevedo, Julia Amanda Rodrigues Fracasso, Marco Aurélio Lucio, Mateus Vidigal de Castro, Marlon Lemos Dias, Francislaine Aparecida dos Reis Lívero, João Tadeu Ribeiro-Paes

**Affiliations:** 1 Human Genome and Stem Cell Research Center, Institute of Biosciences, University of São Paulo—USP, São Paulo 01246-904, Brazil; lincolnmoro@usp.br (L.G.M.); mateusvcastro@gmail.com (M.V.d.C.); 2 Department of Genetic, Ribeirão Preto Medical School, University of São Paulo—USP, Ribeirão Preto 14040-904, Brazil; lucasguarnier@usp.br; 3 Institute of Biomedical Sciences, University of São Paulo—USP, São Paulo 01246-904, Brazil; mauricio.fogaca@usp.br; 4 School of Dentistry, São Paulo State University—UNESP, Araçatuba 16015-050, Brazil; j.fracasso@unesp.br; 5 Graduate Program in Environment and Regional Development, University of Western São Paulo, Presidente Prudente 19050-920, Brazil; marco.aurelio78@hotmail.com; 6 Precision Medicine Research Center, Carlos Chagas Filho Biophysics Institute, Federal University of Rio de Janeiro—UFRJ, Rio de Janeiro 21941-630, Brazil; marlonlemos@biof.ufrj.br; 7 Laboratory of Cardiometabolic Pharmacology, Federal University of Paraná—UFPR, Curitiba 80035-050, Brazil; francislaine@ufpr.br; 8 Laboratory of Genetics and Cell Therapy (GenTe Cel), Department of Biotechnology, São Paulo State University—UNESP, Assis 19806-900, Brazil

**Keywords:** cell culture, 2D cell culture, 3D cell culture, organoids, spheroids, organs-on-a-chip

## Abstract

This comprehensive overview of the historical milestones in cell culture underscores key breakthroughs that have shaped the field over time. It begins with Wilhelm Roux’s seminal experiments in the 1880s, followed by the pioneering efforts of Ross Granville Harrison, who initiated groundbreaking experiments that fundamentally shaped the landscape of cell culture in the early 20th century. Carrel’s influential contributions, notably the immortalization of chicken heart cells, have marked a significant advancement in cell culture techniques. Subsequently, Johannes Holtfreter, Aron Moscona, and Joseph Leighton introduced methodological innovations in three-dimensional (3D) cell culture, initiated by Alexis Carrel, laying the groundwork for future consolidation and expansion of the use of 3D cell culture in different areas of biomedical sciences. The advent of induced pluripotent stem cells by Takahashi and Yamanaka in 2006 was revolutionary, enabling the reprogramming of differentiated cells into a pluripotent state. Since then, recent innovations have included spheroids, organoids, and organ-on-a-chip technologies, aiming to mimic the structure and function of tissues and organs in vitro, pushing the boundaries of biological modeling and disease understanding. In this review, we overview the history of cell culture shedding light on the main discoveries, pitfalls and hurdles that were overcome during the transition from 2D to 3D cell culture techniques. Finally, we discussed the future directions for cell culture research that may accelerate the development of more effective and personalized treatments.

## 1. An Overview: The Cell Culture History

Cell culture entails a spectrum of techniques that facilitate the in vitro development of cells, whether of animal or plant origin, thereby isolating them from their native biological context. This approach seeks to partially recapitulate the physicochemical conditions prevailing in the cell’s original microenvironment. The art and science of cell culture have enjoyed a rich history, finding application across diverse realms within the biological and biomedical sciences, underscoring their profound methodological significance in dissecting cellular responses to distinct biophysical and biochemical stimuli [1,2].

The beginning of cell culture finds its roots in the fields of embryology, biological development, and later, the study of cancer. The earliest documented pursuits can be traced to the late 19th and early 20th centuries. In the 1880s, the renowned physician and microbiologist Robert Koch, then associated with the University of Berlin (Dahlem, Germany), considered the founder of medical bacteriology, was responsible for important contributions regarding the refinement of microorganism cultivation techniques, especially with regard to the identification of the pathogenic agents that cause tuberculosis (Mycobacterium tuberculosis) and cholera (Vibrio cholerae). These discoveries deservedly earned Koch the Nobel Prize in medicine, awarded to him in 1905. In addition, Dr. Koch proposed a methodological approach widely used to assess the causal relationship between microorganisms and infectious diseases. This approach was called “Koch’s Postulates”. Some researchers, however, consider the nomenclature “Koch’s Postulate” to be strictly incorrect, since Koch would have only improved the postulate previously proposed by his mentor Friedrich Gustav Jakob Henle [3,4,5].

Despite this debate, Koch’s most significant contribution to the cell culture knowledge was when he employed gelatin to solidify the culture media. The goal was to facilitate its distribution throughout the culture flask, achieving a consistent and uniform membrane-like layer that covers the whole culture flask surface. This method marked a pivotal enhancement for isolating, identifying, and cultivating individual species of microorganisms in a dish, and represents a cornerstone even nowadays. Furthermore, it is important to highlight that Dr. Koch rigorously emphasized the necessity of sterilizing laboratorial items to minimize or eliminate the possibility of sample contamination, thereby ensuring a high accuracy in experimental results [5,6,7].

Moreover, it is worth mentioning Koch’s studies conducted in collaboration with Richard Petri, during their time working together in the Imperial Health Office (Berlin, Germany). Dr. Richard Julius Petri was a German physician and bacteriologist who made significant contributions to microorganism cultivation techniques as Koch’s assistant. Alongside Dr. Koch, Dr. Petri stablished the so-called “Petri’s dish”, an ubiquitous laboratory apparatus widely used by researchers across the world as a culture flask for microorganisms and/or cell cultivation. Although the nomenclature refers exclusively to Richard Petri, it is important to highlight that the Petri dish was a result of an improvement in the culture dish that Dr. Koch was already using in his research, including those works conducted in partnership with Dr. Petri. However, more than a century later, there is still controversy concerning the nomenclature “Petri’s dish” and its true creator [5,8,9].

Still in the 1880s, Wilhelm Roux, a pioneering German experimental embryologist from the University of Halle (Halle, Germany), embarked on groundbreaking experiments involving embryonic cells extracted from avian sources. His work yielded compelling evidence that it was feasible to sustain cellular life beyond the confines of the host organism by immersing them in a saline solution [10].

Shortly thereafter, the notable legacy of Leo Loeb, a German medical practitioner who later migrated to the United States, came to the fore. His decision to leave Germany was motivated by his dissatisfaction with the nation’s nationalistic and militaristic situation. At the Washington University (Seattle, WA, USA), Loeb emerged as a distinguished experimental pathologist whose groundbreaking contributions in cell culture, transplantation, and hormonal research left an indelible mark on the landscape of medical science [11,12,13,14,15,16,17]. Loeb’s profound dedication to research, combined with his visionary approach to humanitarianism, firmly established him as a pivotal figure in the annals of experimental pathology. His enduring influence continues to inspire and guide scientists worldwide, providing the foundational framework for numerous scientific breakthroughs and advancements in the field.

In 1906, the researcher Ross Granville Harrison (Johns Hopkins University, Baltimore, MD, USA) developed pioneering experiments that laid the foundation for cell culture as we know it today. His investigations focused on growing tissue samples in test tubes. Harrison’s primary focus was on the study of developing nerve fibers in frogs, where he maintained organ fragments in test tubes containing a liquid medium composed of blood clots, saline solution, and agar [18]. Furthermore, Harrison played a crucial role in the development of the “hanging drop” technique, which involved culturing cells within plasma on the underside of glass slides, creating droplets where the cells gathered. This innovative approach, later validated, continues to be employed in contemporary research, evolving through time with refinements and adaptations [19].

Harrison’s notable work culminated in his publication titled “Observations on the Development of Living Nerve Fibers” [18]. In this work, he successfully observed the in vitro development of nerve fibers from a single cell or a cluster over a defined period. However, his research faced a persistent challenge in the form of bacterial contaminations, prompting him to introduce aseptic methodologies. This included the sterilization of surgical materials and the heating of experimental glassware, enabling the conduct of experiments and cell cultivation for extended periods, up to 5 weeks [2,20].

**Figure 1:**
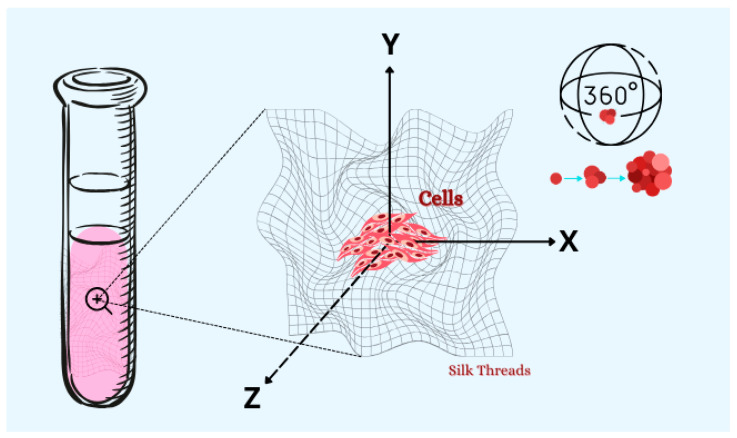
Schematic representation of cells growing around the silk threads impregnated with plasma: the first description of a three-dimensional cell culture, as pioneered by Carrel and Burrows (Adapted from [16]).

Another luminary in the field of cell culture is Alexis Carrel (Rockefeller Institute, USA), a Nobel laureate in medicine in 1912, acclaimed for his introduction of sutures in surgical procedures [21]. Carrel built upon Harrison’s pioneering work from 1906 by developing a method for culturing cells in hanging drops, utilizing glass plate covers. During his investigations, Carrel observed that cells proliferated beyond the confines of the tissue and could be sequentially transferred and manipulated onto new plates. These experiments led to the conception of the “Carrel Flasks”, which served as the precursor to contemporary cell culture flasks [22]. Subsequently, while cultivating cardiomyocytes in chicken plasma, Carrel noted that the interaction between cells and the culture medium was directly linked to increased cell proliferation. However, he also discerned that the region closer to the center of the culture exhibited a higher likelihood of necrosis development. To address this challenge, the researcher cultivated tissue fragments on silk threads saturated with plasma, creating a surface where all cells had uniform access to the available nutrients within the culture medium [23]. For the first time, a detailed description of a three-dimensional cell culture was presented (Figure 1).

Also, the fruitful and important partnership between Alexis Carrel and Charles Lindbergh should be highlighted [21]. Lindbergh was responsible for developing methods to separate blood serum from the rest of the blood and for introducing the use of glassware known as “Pyrex Glass” for cell cultivation. The flasks had the crucial advantage that they were resistant to high temperatures, enabling sterilization in autoclaves, and maintaining temperatures between 120 and 170 °C. Carrel consistently emphasized the need to use sterile materials [22,23], an important consideration, since these experiments were conducted before Alexander Fleming discovered the first antibiotic. Around the 1930s, Carrel and Lindbergh published studies describing technologies that supported many experiments until the 1980s, when more sophisticated growth factors, cytokines, and complex culture media were introduced, characterizing the technologies currently used worldwide [24,25,26,27,28,29].

One of Carrel’s notable contributions was the isolation and cultivation of one of the first immortalized cell lines derived from chicken embryonic hearts [22,23]. This was only possible due to the adoption of a strict sterilization methodology and consecutive changes in the culture media involving washing with Riger’s solution. This strain underwent hundreds of passages and was maintained until mid-1964, when it was finalized 2 years after Carrel’s death. The strain described and cultivated by Carrel generated significant interest at the time, and it was established that the cells could survive indefinitely.

The immortalization of cell cultures can be induced by factors such as oncogenic viral infections, radiation, and carcinogenic substances, and has been observed in various cultures throughout the 1940s and 1960s. One notable example of immortalized cells is HeLa. These cells, which have become fundamental in scientific research, originated in 1951 when Henrietta Lacks was diagnosed with aggressive cervical adenocarcinoma at the Johns Hopkins Hospital in Baltimore (Baltimore, MD, USA). After performing a cervical biopsy, the samples were sent to Dr. George Gay, Director of the Tissue Culture Laboratory [2,20]. Mary Kubicek, his assistant, noticed that the cells remained viable in a nutrient solution based on chicken plasma and cultured Henrietta Lacks’ specimen, resulting in robust, rapidly dividing cell cultures. This remarkable cell line was named HeLa, abbreviated as the initial letters of the patient’s name (Henrietta Lacks). It is worth mentioning that more than 70 years later since their isolation, HeLa cells still survive, which is more than twice the lifespan of Henrietta, who passed away in October 1951 at the age of 31 [2,19,30].

After Carrel’s pioneering work, approximately 35 years had passed before other researchers began to investigate and improve cell culture techniques. Notable scientists, such as Johannes Holtfreter, Aron Arthur Moscona, and Joseph Leighton, contributed in the advancing and refining of cell culture techniques [31].

In the field of developmental biology, Johannes Holtfreter, from the University of Heidelberg (Heidelberg, Germany), described an innovative method that allowed the formation of spherical cell aggregates to prevent cells from adhering to the surface of the culture flasks, thus promoting the tridimensional development of these cells. Later, Holtfreter further refined the techniques previously used by introducing an apparatus that agitated the culture flasks. This facilitated contact between the cells and promoted the diffusion of the surrounding nutrients [32,33].

Another notable researcher in the field of developmental biology, Aron Arthur Moscona (University of Chicago, Chicago, IL, USA), made several contributions to refining cell culture techniques. Initially, studies on avian embryonic cells showed that cells from distinct organs did not assemble as a mixed structure [34]. Furthermore, in a subsequent investigation, Moscona designed an experiment where cells derived from the lungs of mice and chicks were cultured into contact, resulting in the formation of cell aggregates after a few days. As a result, Moscona obtained liver and cartilage tissues in vitro. This pioneering work positioned Moscona at the forefront of research on cellular chimeras [35]. In addition, Moscona introduced a technique for cultivating cells using Erlenmeyer flasks under constant agitation. The continuous shaking of the culture flasks was intended to prevent the cells from adhering to the surface while stimulating the formation of cell aggregates in a three-dimensional configuration [36].

Back in the 1950s, Joseph Leighton (University of Princeton, USA), a specialist in histology and cellular pathology, raised a crucial concern about maintaining cellular tissue architecture during development in culture flasks. He noted that despite the remarkable importance of two-dimensional (2D) cultures, this technique had significant limitations, especially with regard to the space available for cell development, which was not in line with the natural development of these cells in vivo [37]. In one of his most innovative studies, Leighton cultivated cells and tissue fragments in a three-dimensional (3D) matrix made up of a cellulose sponge saturated with plasma obtained from bird embryos. This system was then inserted into a culture flask containing nutrients and subjected to constant agitation. As a result, the study revealed that the 3D arrangement of the cellulose sponge matrix allowed the cells to proliferate and migrate in all directions, more accurately reproducing the behavior of these cells in their organs of origin (in vivo). In addition, these 3D cultures had a significantly larger cell surface area when compared to 2D cell cultures [38]. Based on Leighton’s pioneering studies, it became clear that there was a distinction between 2D and 3D cell culture methods, with 3D cell culture systems standing out for their advantages, including greater fidelity in reproducing in vivo cellular development and behavior [31,37].

In the early 1960s, Ernst McCulloch (University of Toronto, Toronto, ON, Canada) and James Till (Ontario Cancer Institute, Toronto, ON, Canada) began a series of experiments involving the injection of bone marrow cells into irradiated mice. The authors observed that small nodules formed in the spleens of the mice, directly proportional to the number of bone marrow cells injected. Till and McCulloch termed these nodules “spleen colonies” and postulated that each nodule originated from a single bone marrow cell, perhaps a stem cell [39,40]. In later work, Till and McCulloch, in collaboration with Andy Becker (undergraduate student) and Lou Siminovitch, from the University of Toronto (Toronto, ON, Canada), published in 1963 two articles that represent fundamental milestones for the consolidation of self-renewal capacity and, as a result, the formulation of the concept of bone marrow stem cells [41,42].

Another important milestone in the history of cell cultures refers to the work developed, from the 1960s onwards, by the Russian physician Alexander Friedenstein (University of Moscow, Moscow, Russia) which represents cardinal contributions in the discovery and establishment of the concept of mesenchymal stromal/stem cells. From bone marrow cell cultures, Friedenstein and collaborators identified and isolated a subpopulation of non-hematopoietic cells, adherent to culture vials, with a fibroblastoid appearance, with the formation of discrete colonies resulting from clonal multiplication, from a single fibroblastic colony-forming cells, the so-called “Fibroblast Colony Forming Cells” (FCFCs) or “colony forming units fibroblastic” (CFU-F) [43,44,45,46,47,48]. In vivo transplantation experiments demonstrated the multipotential nature of CFU-F, since it was possible to obtain different lineages of mesenchymal/mesodermal origin (osteocytes, chondrocytes, and adipocytes) from a single stromal cell [49,50]. Cells of mesenchymal origin were later named “marrow stromal stem cells” by Maureen Owen (University of Oxford, Oxford, UK) [51,52] and subsequently, as proposed by Arnold Caplan in 1991, the term “mesenchymal stem cells” (MSCs) was adopted [53]. In 2005, the International Society for Cellular Therapy (ISCT) proposed that the scientific community adopt, in all written and oral communications, the nomenclature “multipotent mesenchymal stromal cells” [54], but variations in the nomenclature still persist in the literature, such as mesenchymal stem cells, mesenchymal stromal cells, and mesenchymal stromal/stem cells. Finally, the work of Friedenstein and collaborators, especially the partnership established with Maureen Owen [48], represented pioneering and seminal contributions opening new perspectives in cell therapy and regenerative and translational medicine.

In 1964, Malcolm Steinberg and Stephen A. Roth, both from the University of Princeton (Princeton, NJ, USA), proposed the adhesion hypothesis, which posited that the cellular rearrangement was influenced by thermodynamic mediators on different adhesion surfaces [55]. However, this hypothesis gained greater significance only in later years, particularly as cells began to be isolated and studied in greater depth, with a focus on stem cells.

Research involving stem cells accelerated from the 1980s onwards, when various researchers were able to isolate and cultivate pluripotent stem cells derived from mouse embryos [56]. In 1981, Martin Evans and Matthew Kaufman, both from the University of Cambridge (Cambridge, UK), reported the establishment of cell lines derived from mouse blastocysts, which could differentiate in vitro or, after inoculation into mice, give rise to tumors with cells originating from the three embryonic layers—teratomas [57]. In the same year, in December 1981, Gail R. Martin (University of California, San Francisco, CA, USA) published an article in which she described “[…] the establishment of cell lines from normal mouse embryos that form teratocarcinomas when injected into mice”. In this work, Martin used the term “embryonic stem cells” for the first time in the literature [58]. It is important to highlight that the establishment of in vitro embryonic stem cell cultures allowed the modification and implantation of these cells in adult females, generating genetically modified mice [59]. Because of these works, Martin John Evans was, together with Mario Capecchi and Oliver Smithies, awarded the Nobel Prize in medicine and physiology in 2007.

About 17 years after the work of Evans, Kaufman, and Martin, James Thomson’s team (University of Wisconsin, Madison, WI, USA) established, for the first time, the cultivation of human embryonic stem cells obtained from the inner cell mass of blastocysts from human embryos on the 5th day after fertilization. These pluripotent cells, which had high differentiation potential across a broad range of tissues, were characterized by their normal karyotypes and high telomerase activity levels, making them useful for various applications in research and medicine [60].

A paradigm shift occurred in 2006 when Kazutoshi Takahashi and Shinya Yamanaka, from the University of Kyoto (Kyoto, Japan), described a method that allowed the reprogramming of already differentiated stem cells, creating the so-called “induced pluripotent stem cells” (iPSCs). Researchers were able to obtain iPSCs from adult fibroblasts and mouse embryonic stem cells using only specific markers and growth factors (Sox2, Oct3/4, Klf4, and c-Myc). The result was that iPSCs exhibited properties and characteristics similar to embryonic stem cells, as well as expressing some of the same marker genes [61,62].

**Figure 2:**
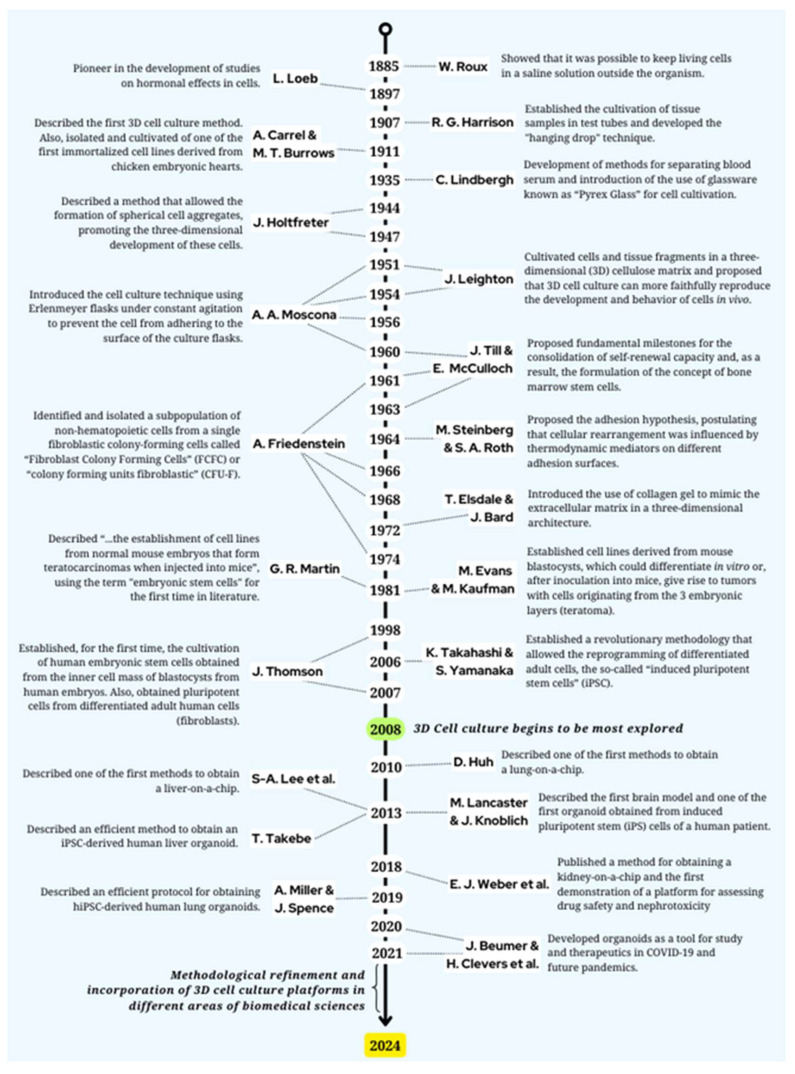
Timeline review of researchers who have contributed to scientific cell culture knowledge and techniques since 1885.

At the same time, in 2007, James Thomson and his team, from the University of Wisconsin (Madison, WI, USA), obtained pluripotent cells from differentiated adult human cells (fibroblasts). However, the authors used a different combination of genes (Oct4, Sox2, NANOG, and Lin28) compared to those used by Yamanaka’s group [63]. Figure 2 provides a chronological overview, spotlighting the eminent researchers who have pioneered and refined cell culture methodologies since 1885 up to nowadays.

With the capability of generating pluripotent stem cells from adult fibroblasts, there has been a substantial increase in the availability of raw materials for research in cellular biology and development. This advance has spurred remarkable progress and the development of previously unimaginable cell culture techniques. Additionally, as somatic cell reprogramming methodologies have become established, various cell types have been effectively utilized in generating iPSCs, expanding beyond fibroblasts. It is notable that peripheral blood cells and urinary cells offer less invasive procurement methods compared to fibroblasts, which typically necessitate skin biopsies. This advantage in accessibility and non-invasiveness underscores the significance of these alternative sources in iPSC derivation. Table 1 showcases the pivotal studies regarding iPSC derivation from diverse cell sources.

## 2. Two-Dimensional (2D) Cell Cultures

Two-dimensional (2D) cell cultures have been widely used in biomedical research. This technique is used to investigate the physiology of cells and tissues under conditions that partially mimic those found in vivo. These investigations cover a range of topics including cell differentiation, migration, growth, physiological mechanisms, and cellular responses to biochemical changes in the microenvironment in which they are cultured [1,71,72]. The 2D cell culture technique is based on the growth of a single cell line on flat, adherent surfaces, such as Petri dishes or culture flasks, containing a supplemented culture medium, and can be applied to a wide range of tissues and cell types [14,73,74,75]. Even for cells that do not naturally adhere to plastic or glass surfaces, such as embryonic stem cells and induced pluripotent stem cells (iPSCs), it is possible to promote adhesion using specific coatings like poly-L-lysine, Matrigel, or fibronectin [76]. This facilitates the cultivation of these cells in a monolayer configuration. Monolayer cell culture methods have some characteristics that make them attractive for research in cellular biochemistry, such as uniform access to nutrients and growth factors present in the culture medium, resulting in homogeneous cell growth and proliferation [77,78,79]. The technology of 2D cell culture has been a crucial tool in biomedical research since the early 20th century, initially focusing on understanding cellular physiological mechanisms. However, over time, various other approaches and applications for this technique have been investigated. It has been widely used in cancer-related studies, although their limitations have led to a gradual decrease in their use in this context. Nevertheless, this technique is widely used in toxicity tests. These tests are crucial for evaluating cellular viability in response to therapeutic candidates and other compounds in general. Additionally, they allow for the determination of the impact of various compounds on genetic material, including genotoxicity and mutagenesis tests [80,81,82,83,84]. The studies employing these approaches have played an important role in reducing the use of animals in research, as suggested by the “3Rs” principles (reduction, refinement, and replacement), proposed by Russell and Burch in their work “The Principles of Humane Experimental Technique”, published in 1959, which addresses and synthetizes a new reflection on the ethical principles relating to the use of animals in scientific research [85].

Cellular toxicity tests have a wide application for therapeutic candidates, as any promising compound should not exhibit significant cytotoxic activity. A classic example of this is the testing of plant-derived compounds that may have potential application as phytotherapeutics. Prior to conducting tests in animal or human models, it is essential to identify potential cytotoxic effects of these substances in vitro during preclinical phases [86,87,88].

Furthermore, new techniques have been developed to enable the simultaneous cultivation of multiple cell types in a monolayer environment, commonly referred to as co-culture, which developed to mimic the in vivo microenvironment more efficiently [89]. These tests are undertaken to investigate potential cellular interactions between different cell lines or to analyze how these lines interact with the surrounding microenvironment and the extracellular matrix (ECM) [90,91]. The applications of these techniques are particularly notable in research involving nervous system cells, where the co-culture of microglial cells with neural stem cells (NSCs) can induce dopaminergic differentiation of NSCs due to the release of differentiation factors. On the other hand, the co-culture of these NSCs with astrocytes promotes their neural differentiation [90].

However, despite the significant and fundamental contribution of 2D cell culture to the advancement of knowledge in various areas of biomedical sciences, this technique has some limitations. One of the main limitations is the lack of contact between cells and the surrounding extracellular matrix (ECM), which can lead to a low fidelity in processes in vivo. This is because cells in vivo have specific structural and morphological characteristics that play a crucial role in cellular physiology [1]. Another limitation is associated with the composition of the ECM used in the cultivation. Some cell lines require a highly complex ECM for proper in vitro proliferation, such as hepatic cell lines, which are surrounded by a highly intricate ECM in the liver. Therefore, the stabilization of these cell lines in monolayer cultures becomes a challenging task due to the complexity in reproducing the microenvironment required for the cells to perform vital functions [92,93,94]. Cellular physiology, in vivo, is influenced by cell morphology and organization, aspects that are impacted in 2D cell culture. This can affect cellular proliferation, differentiation, apoptosis, protein expression, and other cellular processes [90]. In this regard, the development of new study models that can reduce the use of animals in research, while allowing for a more faithful representation of in vivo conditions in vitro, becomes an important step forward for the advancement of therapeutic efficacy tests, pathophysiology, and tests of new drugs [21,95,96,97]. To overcome some of the inherent limitations of 2D cell culture, a more complex cell culture methodology has been increasingly explored as an alternative method to mimic in vitro the behavior of tissues in vivo: the three-dimensional (3D) cell culture.

## 3. Three-Dimensional (3D) Cell Culture: Spheroids and Organoids

Three-dimensional (3D) cell culture models, such as spheroids and organoids, complement and offer some new perspectives on two-dimensional (2D) cell cultures [26,27,98,99,100,101,102,103,104,105,106,107]. This technology is currently considered a highly promising alternative for use in conjunction with animal models and 2D cell culture, allowing for a reduction in the use of these models in basic research [26,28,29,104,108,109,110,111,112]. These models allow the simultaneous cultivation of different cell types enabling the replication of both cell–cell and cell–ECM interactions. In addition, it can mimic the characteristics of the organ or tissue from which the cells are derived, including gene expression, cell proliferation, differentiation, migration, and metabolic functions [93,96,113,114,115,116].

The importance of 3D cell cultures was initially highlighted in 1970 when Robert Sutherland, from the Ontario Cancer Treatment and Research Foundation (London Clinic, London, ON, Canada) and the Departments of Therapeutic Radiology and of Surgery (University of Western Ontario, London, ON, Canada), and his colleagues developed multicellular spheroids to recapitulate the functional phenotype of human tumor cells and their responses to radiotherapy [117,118]. In a pioneering study, Tom Elsdale and Jonathan Bard, from the Western General Hospital (Edinburgh, Scotland), introduced the use of a collagen gel as a scaffold for fibroblast culture. The objective was to provide a three-dimensional substrate architecture that supported cell proliferation. At the end of the study, it was observed that unlike conventional 2D cell culture, the collagen gel partially mimicked key features of the in vivo extracellular matrix, allowing cells to proliferate in a three-dimensional environment, an essential condition for tissue development and differentiation [119]. Shortly after, in 1977, Anne Hamburger and Sydney Salmon (University of Arizona, Tucson, AZ, USA) obtained a three-dimensional culture using a soft agar solution, demonstrating that the morphology and behavior of cells growing in a tumor mass and under 3D conditions showed remarkable similarities [120]. In the same year, Matrigel was introduced as a basement membrane extracellular matrix extracted from mouse sarcoma tumors, containing a unique mix of ECM components and growth factors [121]. This preparation became fundamental for supporting in vitro cell cultures, enabling the growth of various cell lines in a three-dimensional conformation. Over the years, Matrigel has established itself as an indispensable tool in the development of 3D cultures, facilitating the mimicry of the complex cellular and structural interactions found in living tissues. The commercial availability of Matrigel and other similar preparations has continuously optimized the culture process, allowing for significant advances in research.

**Figure 3:**
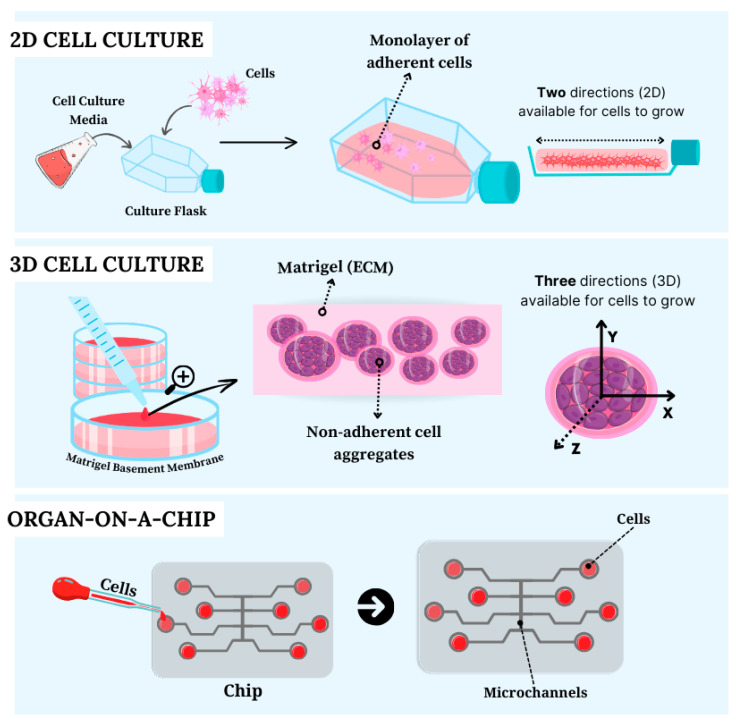
Schematic representation of cell culture techniques used as experimental models in research: two-dimensional (2D) cell culture, three-dimensional (3D) cell culture, and an organ-on-a-chip basic design.

More specifically, spheroids represent the basic units of tissue engineering capable of mimicking the events that naturally occur during embryogenesis, morphogenesis, and organogenesis. Spheroids consist of the cultivation or co-cultivation of any type of primary cell lines, adult stem cells, or iPSCs, that self-organize into three-dimensional architectures, either spontaneously or through external stimuli, forming small spherical cellular aggregates without the need for a predefined culture substrate for cells to adhere to (Figure 3) [122,123].

Currently, several methods exist to produce spheroids and they can vary from the hanging drop technique to the use of magnetic levitation [105,124,125,126,127]. Spheroids are used as models in disease studies, drug screening, and the identification of potential new targets and therapeutic candidates [25,107,128,129,130,131]. The spherical structure of spheroids leads to the formation of gradients of nutrients, lactate, oxygen, carbon dioxide, and pH. These gradients significantly influence cell proliferation, with more proliferative cells found on the outer surface, quiescent or senescent cells in the middle, and apoptotic cells in the inner regions creating a necrotic core [132]. It is important to mention that the necrotic core is considered a key limitation for several authors in the literature. On the other hand, this characteristic makes it an ideal model for studying various types of cancer, due to its similarity to the environment found in cancer cells [27,112,129].

Although showing similar characteristics, organoids are more complex 3D systems than spheroids. Currently, there are different definitions of organoids. Initial scientific interpretations have defined organoids as structures derived from stem cell clusters that self-organize and self-renew through cell–cell and cell–ECM interactions to mimic organogenesis in vitro. Some studies have shown that organoids can also be created using differentiated cells [133,134]. Organoids can be obtained by combining iPSC- and adult tissue-derived stem cells (such as adipocytes and bone marrow cells) and can be differentiated into various human cell types, in addition to being derived from primary culture cells, isolated directly from the target tissue [135]. However, these 3D systems present characteristics such as self-organization, multicellularity, and functionality, with the potential for cell differentiation and self-organization mediated by the complexity of the culture medium, strict environmental control, and the addition of growth and differentiation factors [28,97,136]. These processes can be clearly evidenced by cerebral organoids, representing a significant advancement compared to 2D culture, where neuronal development was limited due to the lack of cell–cell interaction and deficit in neuronal self-organization. Different strategies have been adopted to generate organoids in which specific cell types are cultivated in solid three-dimensional scaffolds derived from natural ECM or biopolymers, ceramics, and metals [122,123,137,138,139], or in suspension on bioreactors [134].

Currently, organoids are used as models in research aimed at identifying and understanding the pathophysiology of various genetic and infectious diseases (including Sars-CoV-2 infection), the mechanisms involved in the development and treatment of tumors, and for the study of new medications and therapies, such as cell therapy, for diseases that lack effective treatment or a reliable cure [28,96,100,123,140,141,142,143]. For instance, hepatic organoids have been employed in the study of cystic fibrosis, liver steatosis, Alagille syndrome, viral hepatitis, sclerosing cholangitis, and alcohol-related diseases [144,145,146].

A critical aspect to be considered is the increasing complexity achieved by organoids. Notably, the most sophisticated liver organoid was developed by Takebe and colleagues in 2013, where different stem cell lines were used to induce not only the three-dimensional formation of differentiated cellular aggregates into liver cells but also in situ vascularization [147]. The vascularization has represented a significant challenge in the development of organoids, as it seeks to achieve the highest possible fidelity to the in vivo tissue.

The cerebral organoid model was pioneered developed by the group of Juergen A. Knoblich (Institute of Molecular Biotechnology, Vienna, Austria) and it was one of the first organoids obtained from iPSCs derived from a human patient. The obtained brain organoid was then used to study a specific type of microcephaly, as an alternative to the difficulty of reproducing this microcephaly in a murine model [148,149]. Since then, cerebral organoids or “mini-brains” have been employed for studying the cellular and molecular bases of various neurological disorders, such as autism, schizophrenia, Alzheimer’s and Parkinson’s disease, and other similar disorders [148]. This approach provides valuable insights into the underlying mechanisms of these conditions and identifies the potential therapeutic targets. Additionally, cerebral organoids enable the investigation of complex processes involved in human brain development, including cell proliferation, neuronal migration, cortical layer formation, and neural circuit establishment [149,150]. However, similar to hepatic organoids, brain organoids also have limitations in terms of model complexity. Notably, the lack of vascularization and absence of immune cells, along with the reduced spontaneous formation of astrocytes and GABAergic inhibitory circuits, pose significant challenges. In this context, the current research is aimed at overcoming these limitations, with the goal of improving the fidelity of the models and their ability to accurately recapitulate human brain physiology.

In addition to brain and liver organoids, models of organs such as the uterus, fallopian tubes, ovaries, and endometrium have been used in the study of diseases related to the female reproductive system, encompassing topics such as endometriosis, endometrial hyperplasia, and carcinomas [114,151,152]. Additionally, organoids representing organs such as the intestine, lungs, and mammary glands have become a focus of investigation [98,100,153,154,155]. Figure 3 summarizes the three cell culture methods used as experimental models.

Organoids and spheroids are promising technologies in regenerative and translational medicine, especially in personalized medicine. In particular, personalized medicine represents a promising approach that can revolutionize the treatment of various diseases, especially those of genetic origin. This area is based on the use of genetic markers, transcriptomics, proteomics, and metabolomics, aiming to individualize preventive and therapeutic methods [28,145,156,157].

Personalized medicine achieves a good standard treatment for each patient by precisely identifying markers [136]. This approach is potentiated by the use of cell culture models derived from the patient’s own cells, enabling a deeper understanding of disease development and the interactions between genetic and epigenetic factors that results in personalized treatments for each patient, including everything from medication administration to the adoption of alternative therapeutic strategies [136,158,159].

However, despite the substantial innovations provided by 3D cell culture models in the biomedical field and the notable impact on reducing the use of animal models, these models still present limitations that render them unable to completely replace traditional study methods [97,108,122]. The primary intrinsic limitation of 3D cell culture models lies in the absence of functional vascularization (presence of blood vessels), which frequently results in immature cellular development, inefficient nutrient distribution, and the formation of necrotic areas in the central nucleus of the culture [113,115]. In this context, it is worth mentioning the pioneering attempt to establish an endothelial cell culture designed by Judah Folkman and collaborators. In his studies of cancer (tumor) growth, Dr. Folkman suggested that endothelial (blood vessel) cells could be cultivated in a three-dimensional system to explore the formation of blood vessels in a more physiologic relevant environment [160,161,162]. Nonetheless, despite excessive efforts, few groups have managed to create a complex model with angiogenesis induction.

In the face of these challenges, research groups have been working on exploring new strategies, such as laser ablation, the use of canalized scaffolds, and the simultaneous cultivation of vascular endothelial cells, with the aim of establishing a microvascular network within the culture scaffolds or in direct contact with the cells [163,164,165]. However, it is essential to highlight that protocols involving the cultivation of vascular endothelial cells still require additional investigations to determine the ideal cultivation conditions in order to establish a standardized method [108,137,166].

Furthermore, the lack of structure organization must be highlighted. Although most of the spheroids and organoids may contain the correct cell types, there is an absence of a structure organization that resembles an in vivo organ architecture. Also, compared to 2D cell culture systems, the 3D methods require considerable time and significant quantities of reagents and materials for proper implementation, without allowing for precise control over the physical–chemical properties of the cellular microenvironment that influence the maintenance of the culture. In this context, the research is ongoing to integrate 3D cell culture with microfluidic systems, known as organs-on-a-chip. Although these systems show promising prospects, they represent a relatively recent technology that still in its initial stages of development [103,167,168].

## 4. Organ-on-a-Chip

The technology of 3D cell culture known as organ-on-a-chip emerges as a promising approach to overcome the inherent limitations of conventional 2D spheroids and organoids [28,136]. This technology is based on microfluidic systems where various 3D cell cultures are maintained in integrated systems, interacting through microtubes and microstructures (Figure 3). These systems have some advantages over traditional cell culture methods, such as the ability to control cell adhesion, provide mechanical stimulation to cells, and allow for tissue perfusion, the creation of artificial vascularization that replicates the characteristics of blood vessels, and the uniform distribution of nutrients to cells. This enables the modelling of complex human organism characteristics in a highly controlled in vitro environment [97,103,136,169].

In 2010, researchers at Harvard University developed the first lung-on-a-chip model. In this pioneering study, Dongeun Huh and colleagues conceived a microfluidic system composed of two separate microchannels separated by a porous polydimethylsiloxane (PDMS) membrane [170]. This membrane was coated with a specific extracellular matrix, and subsequently, human alveolar epithelial cells and pulmonary microvascular endothelial cells were cultured on opposite sides of the membrane. As the cells reached adequate confluence, an air–liquid interface was achieved firstly by removing the fluid on the upper channel, and then applying vacuum to the sides of the culture compartment, with the purpose of reproducing the biomechanical forces associated with respiratory movements and thus mimicking the natural functioning of the lung more precisely. The results of this study demonstrated that the system allowed for simulating cellular responses to pulmonary bacterial infections, evaluating pulmonary inflammatory responses, and conducting toxicity studies of compounds, highlighting the potential of these microfluidic systems as viable alternatives to traditional animal tests [71,136,170].

Subsequently, from a similar design, other organ-on-a-chip models were developed to evaluate the toxicity of different compounds, investigate the potential of new medications or therapeutic targets, and model diseases [171,172]. A notable example is the heart-on-a-chip model developed by Kevin E. Healy and his collaborators at the University of California (Berkeley, CA, USA), based on the differentiation of human iPSCs into cardiomyocytes, known as the microphysiological cardiac system (MPCS). In this study, the researchers evaluated the cellular responses of the developed model in the presence of pharmacological agents with known clinical effects, such as Isoprenaline, E-4031, Verapamil, and Metoprolol, and compared the results with the pharmacological responses of these drugs in traditional cell culture models. After 24 h of culture, it was observed that the cells in the system presented spontaneous contractions (with a frequency of 55~80 bpm), a vital characteristic for mimicking the behavior of a natural heart. The results demonstrated that the system is highly effective as a versatile study model with various applications in the pharmaceutical industry, as well as in developmental biology studies. This study highlighted the superiority of the MPCS compared to traditional 2D cell culture models used for similar purposes [173].

In the following year, a brain-on-a-chip model was developed by researchers from the Johns Hopkins University (Baltimore, MD, USA) and Yale University (New Haven, CT, USA). In this work, Andre Levchenko and his colleagues aimed to elucidate and understand the mechanisms involved in the migration of neural progenitor cells (NPCs) in the central nervous system when stimulated by chemoattractants. Using a silicone elastomeric device, the researchers induced the differentiation of human pluripotent cells into glial and neural cells to replicate the microenvironment of the central nervous system. This model allowed for the mimicking and evaluation of cellular interactions between NPCs and the brain tissue, providing valuable information about processes still not fully understood. The authors highlighted that the brain-on-a-chip model represents a promising and convenient tool for studies related to neurological development, neural oncology, toxicology, and neural regeneration [174].

In 2018, Elijah Weber and colleagues, from the University of Washington (Seattle, WA, USA), established a kidney-on-a-chip model by culturing primary human proximal tubular epithelial cells (PTECs) in a microfluidic chip system. They successfully evaluated the nephrotoxicity of polymyxin B, a polypeptide antibiotic, along with two structural analogues, NAB539 and NAB741. When the cells were exposed to polymyxin B, a significant increase in renal damage signals and cholesterol biosynthesis was observed. However, minimal changes were observed when the cells were exposed to the analogues of polymyxin, demonstrating the preclinical safety of NAB741 and NAB739 [175].

On the other hand, in 2019, Jang and colleagues developed various liver-on-a-chip models using sinusoidal endothelial cells from the liver, as well as primary human, rat, and canine hepatocytes. The researchers aimed to evaluate the hepatotoxic effects induced by bosentan, a receptor antagonist of endothelin, a compound known to cause cholestasis in humans but not in rats and dogs. Initially, it was observed that the liver-on-a-chip models produced albumin, a characteristic protein of liver cells. It is important to highlight that this complex system was based on the first liver microfluidic system, developed in 2002 at the Massachusetts Institute of Technology, in Cambridge, by Powers and colleagues, which paved the way for the advancement of more modern liver-on-chip technologies [176]. The results demonstrated that these systems could detect and mimic not only hepatic toxicity phenotypes but also conditions such as steatosis, cholestasis, fibrosis, and liver cell lesions. In summary, the authors emphasized that microfluidic chip systems provide powerful tools for a better understanding and prediction of liver toxicities, lesions, and diseases compared to traditional cell culture methods [177].

Also in 2019, researchers at the Cincinnati Children’s Hospital Medical Center (Cincinnati, OH, USA) developed a pancreas-on-a-chip model by culturing epithelial cells derived from pancreatic ducts and islets in a single microfluidic system. The purpose of this study was to evaluate the functional relationship between these two types of cells in patients diagnosed with cystic fibrosis, a genetic disease associated with dysfunction of the cystic fibrosis transmembrane conductance regulator protein. Additionally, the researchers investigated the relationship between cystic fibrosis and other pancreatic dysfunctions, such as the development of diabetes. The results indicated that attenuation of the cystic fibrosis transmembrane conductance regulator protein led to a reduction of approximately 50% in the amount of insulin secreted by pancreatic islet cells, a crucial discovery for understanding the development of diabetes in these patients. The authors highlighted that the pancreas-on-a-chip model can be a valuable tool in diagnosing diseases like diabetes, identifying new therapeutic targets, and promoting personalized medicine for treating this condition [178].

## 5. New Perspectives for Cell Culture Methodologies

**Figure 4:**
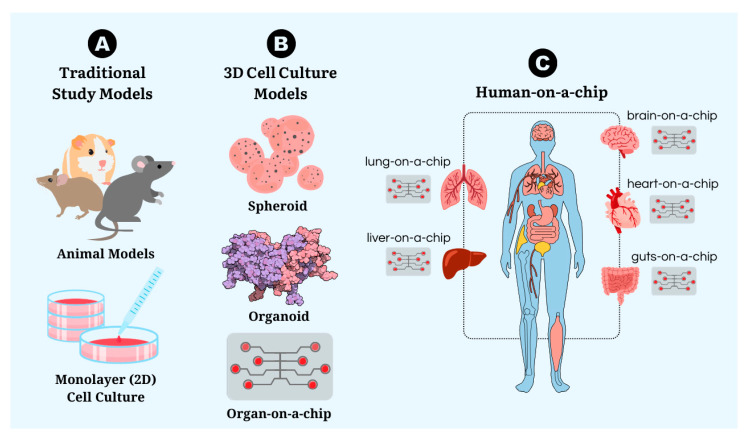
(**A**) Traditional and (**B**) 3D cell culture research study models. (**C**) A schematic representation of the association (connection) of individual organs-on-a-chip for the development of a human-on-a-chip.

Animal models (Figure 4A), which are still the standard and minimum requirement for pre-clinical to clinical stages, have some limitations. These models, along with more advanced in vitro models like spheroids and organoids (Figure 4B), have their own set of drawbacks. To overcome the challenge of reducing animal experimentation, the key is to find a method that can simulate interactions between biological systems without the inherent limitations of animal models.

The increasing complexity of cell culture systems aims primarily to replicate human physiology with the maximum possible fidelity, both in the context of normal development and in the pathophysiology of diseases. These advances are critically important in technical applications such as drug screening and personalized medicine. Consequently, various studies are being developed to obtain a cell culture model that mimics even more complex systems, improving the imitation of human physiology. In particular, microfluidic technology has provided numerous possibilities, including the integration of multiple organs-on-a-chip to simulate interactions between entire systems. However, as any technology in its initial stages of development, organ-on-a-chip models still present some limitations that need to be carefully addressed and evaluated before a standardized method for their acquisition and application can be established [95,99,101,113].

Also, the absence of a universal cell culture medium represents a significant challenge for 3D culture systems, particularly in terms of co-culture methods. Each cell type requires particularities during cultivation and different cell medium compositions [179]. In some cases, the choice of a specific medium can stimulate certain cell types instead of others and impact their reliability, physiological response, and applicability in multiorgan studies [180]. In particular, this can lead to suboptimal performance of organ-on-chip models in the context of organ-on-chip technologies. Considering that many of these models have been used for drug testing and disease modelling, addressing this pitfall is essential for the establishment of standardized 3D conditions, reducing bias, and decreasing costs with multiple media formulations [181,182].

Integrative models of multiple organs-on-a-chip, also known as human-on-a-chip or body-on-a-chip show great promise. This approach makes it possible to recreate the physiology of the entire body by connecting different organs via microchannels. This methodology aims to simulate a human organism and its complex system interactions in a single microfluidic device. In addition to incorporating essential biological barriers, these systems overcome one of the main limitations of conventional 3D cultures, which is the lack of adequate vascularization and gas exchange. In essence, the human-on-a-chip is a convergence of various organ-on-a-chip models, making the method even more complex to simulate nutrient and oxygen exchange between different organ systems (Figure 4C). This represents a notable advancement in biomedical research, offering the ability to study systemic interactions and evaluate the impact of treatments in a more realistic context, without the need for animal experimentation.

However, there are a number of technical difficulties in using this model. These include the need to establish microchannels with very small diameters that are representative of blood vessels and the need to mimic the physicochemical properties to which endothelial barriers are normally subjected. Although challenging, overcoming these hurdles will be essential to investigate physiological and pathophysiological mechanisms using multiple organs-on-a-chip [183,184]. The continuous development of this technology promises to revolutionize preclinical research and accelerate progress towards more effective and personalized treatments. The main applications of experimental models in preclinical trials, as well as the main differences, are described in Table 2.

## 6. Conclusion Remarks

It is unquestionable that 2D cell cultures have played a critical role in the development of science. It is also important to emphasize that a large part of our knowledge about human diseases, drug development, and therapeutic applications is mainly due to the use of 2D cell culture alongside animal models. However, these models still present limitations that have hindered the translation of data obtained in basic research into clinical trials. Currently, various tridimensional cell culture models (3D) are being developed, evaluated, and utilized as study platforms in the fields of biotechnology and biomedicine. This technology enables the reproduction of complex biological characteristics and mechanisms in a highly controlled in vitro system showing significant potential in preclinical studies, new drug screening, and therapeutic applications. In this way, 3D cell culture platforms have in recent years complemented and expanded the research perspectives and enabled great advances in basic and applied research, reducing the gap between preclinical studies and their potential application in regenerative and translational medicine.

## Figures and Tables

**Table 1:** Key studies on the derivation of induced pluripotent stem cells (iPSCs) from various cell sources.

Cell Source	Cell Lineage	Species	Factors	Methods	References
Embryonic/Adult Fibroblasts	MEF and TTF cells	Mice	Oct3/4, Sox2, c-Myc, and Klf4	Retroviral vector transduction; Plat-E cell line expansion	[62]
Adult Fibroblasts	MSC derived from human OCT4 knock-in ES cells	Human	Oct4, Sox2, NANOG, and Lin28	Lentiviral vector transduction 293FT cell line expansion	[63]
Keratinocytes	Keratinocytes foreskin-derived	Human	Oct4, Sox2, c-Myc, and Klf4	Retroviral MSCVpuro vector transduction; Phoenix Amphotropic cell line expansion	[64]
Peripheral Blood Cells	CD34+ mobilized human peripheral blood cells	Human	Oct4, Sox2, Klf4 and c-Myc	Retroviral vector transduction; 293T cells line expansion	[65]
Cord Blood Cells	Human cord blood (CB)-derived endothelial cells (ECs)	Human	Oct4, Sox2, NANOG, and Lin28	Lentiviral vector Addgene transduction	[66]
Amniotic Membrane MSC	MSC placenta derived	Human	Sox2, Klf4, Oct4, and c-Myc	pMX-based retroviruses transduction	[67]
Dental MSC	MSC Human third molar-derived	Human	Oct3/4, Sox2, c-Myc, and Klf4	EcoRI site of the pMX-based retroviruses transduction	[68]
´Renal Epithelial Cells	RPTE cells	Human	Oct4, Sox2, Klf4, and c-Myc	Adgene retroviral vector transduction, HEK293T cell expansion	[69,70]

**Table 2:** Multidimensional analysis of experimental models in biosciences.

	Animal Model	2D Cell Culture	3D Cell Culture	Organ-on-a-Chip	References
Ease of Maintenance	Low (Specialized facilities, handling costs, ethical considerations, daily care)	High (Easy setup in incubators, minimal monitoring)	Moderate (Specific culture media, regular monitoring, skilled handling)	Moderate (Requires microfluidic expertise, precise media control, maintenance of flow rates)	[1,185]
Recapitulation of Biology Development	High (Whole-organism physiology)	Low (Limited interaction with ECM, artificial environment)	Moderate-High (Mimics cell–cell and cell–matrix interactions, but lacks full tissue complexity)	High (Tissue structure, microenvironment, and fluid flow for realistic responses)	[71,186]
Length of Experiments	Long (Weeks to months; e.g., rodent models for cancer studies)	Short (Days to weeks for most cell lines)	Short-Moderate (Days to months, depending on cell type, e.g., organoid development)	Short-Moderate (Hours to weeks; liver-on-chip models can be maintained for weeks)	[77,187]
Genetic Engineering	Low (Gene editing is time-consuming and costly; CRISPR/Cas9 protocols can take weeks to months)	High (Easily transfected, edited; turnaround within days to weeks)	High (Organoids can be genetically modified to mimic disease states)	High (Engineered microenvironments can simulate genetically modified conditions)	[188,189,190]
Physiological Complexity	High (Whole-organism level; includes immune, metabolic, and systemic responses)	Low (Monolayer cultures lack tissue and organ-level complexity)	Moderate (3D structure and cellular diversity, e.g., hepatic organoids mimic liver zones)	High (Can incorporate multiple cell types, ECM components, dynamic flow, real-time monitoring)	[71,191,192,193]
Relative Cost *	Very High (>USD 50,000/year for animal housing, care, and ethical compliance)	Low (<USD 1000 for basic culture setup, ~USD 10 per flask of cells)	Moderate (Varies: USD 5000USD 20,000 per project; specialized reagents and matrices)	High (>USD 10,000 per chip setup, costs increase with custom microfluidics and perfusion systems)	[1,71,190,194,195]
Recapitulation of Human Physiology	Moderate (Species differences affect translation; ~20% success rate in translating findings to human trials)	Low (Lacks tissue organization and complexity, ~8% success in drug discovery translation)	Moderate-High (Better simulation of human tissues; organoids can replicate disease mechanisms)	High (Mimics organ-level responses, fluid flow; improved drug toxicity prediction accuracy of >70%)	[1,187]

Legend. * While it is challenging to determine exact costs for each experimental model due to variability in research design and infrastructure, the values provide approximate estimates to facilitate comparison. These figures are derived from general reports, academic articles, and industry insights into typical maintenance costs for animal models, 2D and 3D cell cultures, and organ-on-a-chip technologies.

## Data Availability

Not applicable.
